# Mindful medical practice: An innovative core course to prepare medical students for clerkship

**DOI:** 10.1007/s40037-020-00591-3

**Published:** 2020-06-05

**Authors:** Tom A. Hutchinson, Stephen Liben

**Affiliations:** 1 Department of Medicine, Faculty of Medicine, Programs in Whole Person Care, McGill University, Montreal, Quebec Canada; 2 Department of Pediatrics, Faculty of Medicine, Programs in Whole Person Care, McGill University, Montreal, Quebec Canada

**Keywords:** Mindful medical practice, Whole person care, Congruence, Experiential learning

## Abstract

**Background:**

Medical students show a decline in empathy and ethical reasoning during medical school that is most marked during clerkship. We believe that part of the problem is that students do not have the skills and ways of being and relating necessary to deal effectively with the overwhelming clinical experience of clerkship.

**Approach:**

At McGill University in Montreal, starting in January 2015, we have taught a course on mindful medical practice that combines a clinical focus on the combination of mindfulness and congruent relating that is aimed at giving students the skills and ways of being to function effectively in clerkship. The course is taught to all medical students in groups of 20, weekly for 7 weeks, in the 6 months immediately prior to clerkship, a time when students are very open to learning the skills they need to take effective care of patients.

**Evaluation:**

The course has been well accepted by students as evidenced by their engagement, their evaluations, and their comments in the essays that they write at the end of the course. In a follow-up session at the simulation centre one year later students remember clearly and enact what they were taught in the course.

**Reflection:**

The next steps will be to conduct a formal evaluation of the effect of our teaching that will involve a combination of qualitative methods to clarify the nature of the impact on our students and a quantitative assessment of the difference the course makes to students’ experience and performance in clerkship.

## Background

### Description of problem and generalizability

There is evidence that rather than experiencing an increase in empathy and ethical reasoning as they progress in their training, medical students actually experience a decrease in these key ways of being [1, 2]. The decline appears to be most severe during their intense clinical exposure during clerkship [3]. One theory is that this decline is a result of being overwhelmed by intimate interactions with patients and other stressful aspects of the clinical milieu [4, 5]. We propose that, although this may be part of the problem, the issue may be fundamentally that students are not given sufficient skills and ways of relating before clerkship that would prepare them to manage effectively their clinical and professional relationships. We believe this is a problem that is general to medical schools across the world but perhaps most immediately relevant to North American medical schools where clerkship can be such a challenging experience for students [4] during the latter part of their medical school trajectory, although there may be differences between Canada and the United States related to a different emphasis on clinical grades in matching for competitive subspecialties.

### The array of potential solutions

There are many potential solutions to this problem including courses in medical ethics, formal mentoring of students, role modelling by teachers, training in empathy and compassion, changing the clinical environment to make it more supportive and nurturing of students, sessions and courses on adaptive expertise, elective courses on healing, relationship-centered care, and mindfulness. All of these can be helpful and most of them have been or are being tried at ours and other medical schools.

## Approach

### Why a particular solution was chosen

At McGill University in Montreal, starting in January 2015, we began to teach a core course for all medical students specifically aimed at training students how to be, and remain, present to their patients and themselves in the intense clinical environment that they would experience in clerkship. The innovative aspects of our approach are: the timing of the course immediately prior to clerkship when students are particularly open to learning skills that will help them take care of patients; the consistent focus on the clinical context in all of our teaching; the clear overall objective of teaching mindful clinical congruence, a combination of mindful presence with a specific focus on ways to be fully present in clinical relationships based on the work of pioneering family therapist Virginia Satir [6]; and the interactive and experiential nature of our teaching.

We are not the only group to teach mindfulness in medical school but one of very few who teach mindfulness as part of the core curriculum [7]. At Monash University a course called the Health Enhancement Program is taught to students in the first year [8] and in Rochester University a course called the Mindful Practice Program is taught primarily in the third year [9]. We have learned from both of these groups. The course we designed would be taught at the end of second year and would be specifically designed to prepare students for their clinical relationships. We believed that leading up to clerkship, students would be very anxious and very open to learning ways to relate more effectively to patients and to themselves in the clinical environment. We designed a 7-session course for small groups of 20 students, who stay in the same group throughout the course, with weekly 2‑hour classes on the topics of: Attention and Awareness; Congruent Communication; Awareness and Decision-making; Clinical Congruence; Building Resilience; Responding to Suffering; Mindful Congruent Practice in Clerkship and Beyond. The group size of 20 was chosen because above that number the challenges of group leadership increase [10]. We chose a 7-week format to fit our curriculum and because we had experience, based partially on the work of Kabat-Zinn [11], of teaching an 8‑week course to healthcare practitioners [12] and elective students on Mindful Medical Practice [13]. The timing of our teaching in the second half of year 2 would coincide for students in the United States with studying for the USMLE step 1 exam which could be seen as both a difficulty and an additional potential benefit of the course.

We have found the combination of mindfulness from the work of Kabat-Zinn [11] and congruence from the work of Virginia Satir [14] a particularly powerful combination to teach students at this stage in their training. Mindfulness teaches students non-judgemental presence in the moment [11], something that they realize is good for their own well-being and that of their patients. Congruence [14] and the communication stances [15] give them a simple way to frame relationships that resonates with their own experience [16] and opens a path to becoming aware of and making a choice about how they are relating and what may be missing in a clinical interaction with a patient [6].

However, it is not just the content of our course that we believe is innovative and impactful. The process is as important or more important. We use a specific arrangement for teaching these classes. Students sit on chairs in a circle without any desk or table in front of them, turn off all cell phones and other electronic devices, and do not take notes in class. Within the framework of each class, which is primarily dictated by the content we aim to teach, we alternate between experiential exercises and conversations with students and between students that invite change [17]. The tutor’s primary role is to listen with focused attention and to ask questions that deepen the conversation in a way that moves the action forward [17]. This creates connections between the tutor and individual students and between students. It is the combination of the content that we teach specifically aimed at promoting mindful congruence in clinical interactions, and the power and depth of the relationships in the classroom that, we believe, will give students the skills and way of being necessary to facilitate their transition to the clinical milieu and blunt the declines in empathy and ethical reasoning that normally accompany the next stage in their training [1–3], a belief that will need to be confirmed by future research.

### Implementation of the innovative solution

We began by writing a proposal for the course based on our previous teaching experience and specifically designed to address key learning objectives listed on our medical school’s webpage [18]. We presented the plan to the Curriculum Committee at a time that a new curriculum was being implemented and there were time slots available at the time we wished to teach the course. We made an overall plan for the topics to be covered and the order in which they would be taught. We relied on individual members of our group to make detailed plans for individual sessions for which they had relevant expertise. Before we taught the course for the first time to medical students we rehearsed each session with a group of 6 faculty and made changes based on that experience and feedback.

## Evaluation

We do not have a formal assessment of whether our teaching changes or blunts the declines in empathy [1, 3] and ethical conduct [2] that the course is designed to address. We have, however, some preliminary information: a) We do have students’ open and enthusiastic engagement and positive assessment of the course as evidenced by their formal evaluations at the end of the course. b) The aspects of the course that students particularly appreciated and wrote about in 18 published essays [16] were mentioned in order of frequency: realizing they were not alone (10^1^); an opportunity to reflect (10); increased self-knowledge (9); benefit of no notes or electronic devises (4); relationship to clinical practice (3); good timing (2); safe environment (2); meditations (2); communication stances (2). c) Students have learned the main concepts that we teach as evidenced by their virtually 100% pass rate on a multiple-choice exam at the end of the course. d) Students retain these concepts and how to enact them in practice as evidenced by their performance in a session at the Simulation Centre [19] approximately 1 year after they have taken the course.

### Sustainability of innovation

We have sustained the teaching of this course over the past 5 years and plan to continue to teach it in the future. It does represent a lot of teaching time for instructors. In the 6 months prior to clerkship, we teach the 7‑week course to groups of 20 students nine times to cover all 180 or so students in one year of our medical school. This means 7 (classes) × 2 (hours per class) × 9 (groups) = 126 h of teaching divided among 6 tutors or roughly 22 h per tutor. Training tutors has been a gradual process with each new tutor sitting in on 7 sessions, co-teaching 7 sessions, and finally teaching 7 sessions on their own or continuing to co-teach. Not every physician can teach this course. The tutors we have chosen have all had training in mindfulness and some regular practice of mindful meditation. They have an ability to facilitate a group of 20 students and an openness to learning the Satir approach to relationships and communication. The biggest challenge we have experienced in teaching this course is identifying suitable tutors. We now have eight tutors trained or in training. We are currently conducting training at other centers with prospective tutors, all of whom are physicians with clinical experience and an interest in mindfulness, using our published detailed course guide [20] as a template for instruction, supplemented by discussions and debriefing with experienced tutors.

## Reflection

We are exploring a qualitative study to learn in a more formal way the nature of the impact of our teaching on our students. This study will include following students through clerkship to determine their practice of what they learned and the barriers to that practice. A direct quantitative answer to the question of whether this teaching has an effect compared with not teaching the course would require a randomized controlled trial. Since the effect is on groups of students, the above trial would probably need to have a cluster design [21]. In the shorter term, we are exploring a stepped-wedge cluster randomization [21] to take advantage of the stepped nature of the teaching in our institution where cohorts of students in the same class are taught the course in sequential 2‑month blocks. In the longer term, we are planning to enrol other medical schools, including in the United States, in a study where the unit of randomization would be a whole medical class and the intervention would be specified by our course guide [20] supplemented by in depth training of tutors. We hope in this way to make a course that we believe is beneficial in preparing students for clerkship available to other centres while at the same time conducting a rigorous study of the course’s effectiveness in helping students relate effectively to patients and blunting declines of empathy and ethical reasoning during clerkship.

### Final thoughts

We believe that the course does provide a clear-cut and tangible way to prepare students for clerkship and to blunt or alleviate the decline in the expression of key values during clerkship. We believe the timing of the course immediately prior to clerkship may be an important factor in its success—students are very curious and committed to learning whatever may help them to do a better job in clerkship.

We learned some key lessons in teaching the course to medical students:

- We were concerned, because we were teaching an unselected group of students who had not volunteered for the course, that there would be some students who were so resistant to this teaching that they would disrupt the process for the whole group. This turned out not to be the case—a very pleasant surprise.
- We have also found that acceptance of the course appears to have increased with succeeding years, possibly because of communication between students from different cohorts.
- One realization in teaching the course which is reflected in our own observations and the students’ essays [16] is that a major benefit of the course is that students get to know each other better and relate to each other on a deeper level. This break down in the sense of isolation that physicians often feel [22] is a major benefit of the course and an important step in contributing to students’ own wellness and preparing them to relate more deeply to their patients.

## Footnotes

1 * number of times mentioned.
